# Assessment of the Relative Performance of the EQ-5D-3L, ICIQ-UI SF and POP-SS Using Data from the OPAL Trial

**DOI:** 10.3390/ijerph19031351

**Published:** 2022-01-26

**Authors:** Linda Fenocchi, Marissa Collins, Andrew Elders, Suzanne Hagen

**Affiliations:** 1 Yunus Centre for Social Business and Health, Glasgow Caledonian University, Cowcaddens Road, Glasgow G4 0BA, UK; marissa.collins@gcu.ac.uk; 2 Nursing, Midwifery and Allied Health Professions Research Unit, Glasgow Caledonian University, Cowcaddens Road, Glasgow G4 0BA, UK; andrew.elders@gcu.ac.uk (A.E.); s.hagen@gcu.ac.uk (S.H.)

**Keywords:** health-related quality of life, EQ-5D, ICIQ-UI SF, POP-SS, urinary incontinence, pelvic floor muscle training

## Abstract

Conducting economic evaluations alongside randomised controlled trials (RCTs) is an efficient way to collect cost-effectiveness data. Generic preference-based measures, such as EQ-5D, are often used alongside clinical data measures in RCTs. However, in the case of female urinary incontinence (UI), evidence of the relative performance of EQ-5D with condition-specific measures such as the International Consultation on Incontinence Questionnaire Urinary Incontinence Short Form (ICIQ-UI SF), measuring severity of UI, and Pelvic Organ Prolapse Symptom Score (POP-SS), measuring severity of prolapse symptoms, is limited. This study employed secondary analysis of outcome measures data collected during the Optimal Pelvic floor muscle training for Adherence Long-term (OPAL) RCT, which compared biofeedback-mediated pelvic floor muscle training to basic pelvic floor muscle training for women with UI. The relative performance of EQ-5D-3L and ICIQ-UI SF, and EQ-5D-3L and POP-SS was assessed for concurrent validity and known-groups validity. Data for 577 women (mean age 48) were available for EQ-5D-3L/ICIQ-UI SF, and 555 women (mean age 47) for EQ-5D-3L/POP-SS. Overall, EQ-5D-3L exhibited very weak association with the ICIQ-UI SF total score, or any subscale. EQ-5D-3L and POP-SS were found to be weakly correlated. EQ-5D-3L was able to distinguish between groups with known differences in severity of UI and also between types of UI. These findings provide useful information to guide researchers in selecting appropriate outcome measures for use in future clinical trials.

## 1. Introduction

Urinary incontinence (UI) affects a large number of women and is often accompanied by negative effects on their health-related quality of life (HRQoL) [1]. It has been suggested that UI occurs in about 20–30% of young women, 30–40% in middle age and up to 50% of women in old age [2]. Conservative treatments such as lifestyle interventions and pelvic floor muscle training offer potential clinical improvements. Assessment of clinical effectiveness should be considered alongside an economic evaluation to assess the cost-effectiveness of an intervention given the scarcity of healthcare resources [3].

Selecting from among the range of outcome measures available to researchers is an important stage in trial design [4]. Condition-specific measures are tailored to specific diseases, and enable a more refined and targeted measurement of treatment effect [5]. Two such condition-specific measures are the International Consultation on Incontinence Questionnaire Urinary Incontinence Short Form (ICIQ-UI SF) and the Pelvic Organ Prolapse Symptom Score (POP-SS). In randomised controlled trials (RCTs) these can be complemented by generic measures used to assess broader impacts on health and wellbeing as a consequence of treatment. The complementary strengths of generic instruments and condition-specific instruments are recognised from guidelines in the United Kingdom (UK), with the most common generic preference-based measure being the EQ-5D [6]. The guidelines advocate the use of EQ-5D together with condition-specific instruments in health assessment [7]. The EQ-5D asks questions about five domains of health (mobility; self-care; usual activities; pain; anxiety and depression), with three levels of response (EQ-5D-3L: no problems, some problems, extreme problems; best 11111, worst 33333) or five levels of response (EQ-5D-5L: no problems, slight, moderate, severe and extreme problems/unable to; best 11111, worst 55555) available [8]. By design, generic preference-based outcome measures produce a single utility score to enable comparison across disease areas as well as between treatments within disease areas. The single utility score obtained can be used in the calculation of quality-adjusted life years (QALY) [9].

The extent to which EQ-5D can usefully assess HRQoL for women with UI is not settled. A study assessing the measurement properties of EQ-5D and the condition-specific measures Symptom Severity Index (SSI) and the Urinary Incontinence-Specific Quality of Life instrument (I-QoL), using data from an RCT of treatment for women with UI concluded that the EQ-5D was not recommended for this population on the basis of low responsiveness to changes in health [10]. Although it would be expected that this clinical population of women have a lower quality of life when compared to the general population, a high level of participants reported full health (i.e., no problems on any dimension) at baseline, reducing the potential for measuring improvements. In contrast, a review of published studies reporting on the psychometric performance of the EQ-5D-3L in people with urinary incontinence concluded that EQ-5D-3L performed well and was appropriate to use in this group [11]. However, no papers were found which assessed the validity of the EQ-5D in relation to ICIQ-UI SF and POP-SS.

This study used data from the Optimal Pelvic floor muscle training for the Adherence Long-term (OPAL) trial to report the comparative performance of generic and condition-specific instruments: EQ-5D-3L, the ICIQ-UI SF and POP-SS. The ICIQ-UI SF is a short form questionnaire that consists of four questions to assess the severity of UI [12]. The POP-SS is a questionnaire with seven questions to assess the severity of prolapse symptoms [13]. The OPAL trial involved 600 women with stress UI or mixed (stress and urge) UI in a RCT to assess the clinical and cost-effectiveness of biofeedback-mediated intensive pelvic floor muscle training compared with basic pelvic floor muscle training [14]. The main trial finding was that there was no evidence that exercise plus biofeedback was better than exercise alone and, therefore, was not recommended to be offered routinely as part of the pelvic floor treatment for women. Specifically, the ICIQ-UI SF and POP-SS measures did not find evidence of a clinical difference, while a cost-utility analysis using EQ-5D-3L indicated that there was not a significant difference in QALYs between the treatment groups at 24 months. As a secondary analysis of the OPAL trial dataset, the relationships between the outcome measures used were evaluated to assess the extent to which EQ-5D-3L correlates with the other measures used, and the ability of EQ-5D-3L to differentiate between clinical groups that are hypothesised to differ. Understanding the measurement properties aids the choice of outcome measure and informs the interpretation of results [15].

Therefore, the aim of this paper was to compare the performance of the EQ-5D-3L in terms of the strength and direction of association (concurrent validity) and differences between known-groups (known-groups validity) to provide evidence of the relative performance of EQ-5D-3L when used in an RCT for women with UI, with ICIQ-UI SF, and/or with POP-SS.

## 2. Materials and Methods

### 2.1. Data

De-identified participant baseline data were available from the OPAL trial from the investigators (AE and SH). The OPAL trial was a multicentre, parallel-group RCT of the clinical effectiveness and cost-effectiveness of biofeedback-mediated intensive pelvic floor muscle training (biofeedback pelvic floor muscle training, providing visual or auditory feedback of internal muscle movement) compared with basic pelvic floor muscle training for treating female stress or mixed set in the UK community and outpatient care settings. Participants were women aged ≥18 years, with new stress UI or mixed UI.

### 2.2. Outcome Measures

The raw data scores for each outcome measure, for each participant, were obtained through the project team. HRQoL was measured using EQ-5D-3L where the respondent is asked to tick which level is most appropriate for their health state on five domains of health (mobility; self-care; usual activities; pain; anxiety and depression). From this, a quality of life score can be calculated ranging from −0.594, indicating a health state worse than death where 0 is death, to 1, indicating full health. The condition-specific measures were the ICIQ-UI SF and the POP-SS. The ICIQ-UI SF calculates a total score, ranging from 0 to 21 from three out of the four questions asked, with recommended severity cut-off scores: slight (1–5), moderate (6–12), severe (13–18) and very severe (19–21) [16]. POP-SS calculates a total score from the seven questions asked, ranging from 0 to 28; the higher the score, the more severe the prolapse symptoms (indicated by higher frequency and number of different symptom types reported) [14].

### 2.3. Data Cleaning and Scoring

From the OPAL trial baseline dataset, two datasets were developed; one for ICIQ-UI SF and EQ-5D-3L and one for POP-SS and EQ-5D-3L. Complete level scores are required to calculate the EQ-5D-3L and full data are required for the domains of the ICIQ-UI SF and POP-SS. Participants with missing data from the ICIQ-UI SF and/or EQ-5D-3L, and the POP-SS and/or EQ-5D-3L, were removed from the analysis. An overall score for each outcome measure per participant was calculated. For EQ-5D-3L the UK tariff was used to calculate a quality of life score for each participant [17]. For ICIQ-UI SF and POP-SS, individual scores for each question were totaled for each participant.

### 2.4. Statistical Analysis

The analysis was undertaken using STATA 13.1 (StataCorp LP, College Station, TX, USA). Concurrent validity and known-groups validity at a single point in time were assessed [18]. Concurrent validity was assessed using Spearman’s rank correlation to determine the strength and direction of the relationship between the variables, as they are measured on an ordinal scale. Both individual question scores and total scores were treated as ordinal measures. The following relationships were assessed: total EQ-5D-3L score and total scores for ICIQ-UI SF; total EQ-5D-3L score and individual scores for the ICIQ-UI SF questions; total EQ-5D-3L scores and total scores for POP-SS; and, total EQ-5D-3L scores and individual scores for the POP-SS questions. Hypotheses were theoretically derived on assumptions about the direction and strength of the relationship between outcome measures. The hypothesis for each outcome measure was that ICIQ-UI SF and POP-SS should have a negative association with the EQ-5D-3L, meaning that decreases in the ICIQ-UI and POP-SS scores (lower scores indicate less symptom severity) are associated with an increase in the EQ-5D-3L score (higher scores indicate better HRQoL). Spearman Rank correlation coefficients are on a scale from −1 to +1. A Spearman Rank correlation coefficient of zero would indicate no association between the variables and a correlation coefficient close to −1 or +1 indicates a stronger association. Spearman Rank correlation coefficients of less than 0.3 are considered very weak, between 0.3 and 0.5 weak, between 0.5 and 0.7 moderate, and greater than 0.7 are considered to be strong [19].

Known-groups validity was assessed using a *t*-test to assess the statistical significance of differences between known sub-groups to analyse the ability of the EQ-5D-3L to distinguish between women with different levels of severity of UI, age and type of UI (stress or mixed). Severity of UI was analysed using two groups: mild/moderate and severe. For the ICIQ-UI, mild/moderate had a score less than 13 and severe a score of 13 plus [16]. For the POP-SS, a similar cut-off was used, with mild/moderate being assigned a score of 13 or less and severe a score of 14 plus [20]. The type of UI was determined prior to the start of the RCT, and these groups were used for the analysis. The most common form of UI is stress which is when involuntary urine leakage occurs due to exertion or effort, for example, when sneezing or coughing. Urgency UI is involuntary urine leakage followed by, or before, a compelling desire to pass urine. Women can have both stress and urge, referred to as mixed UI. Based on the original trial analysis, for the age of participants, the data were split into those aged less than 50 years and those aged 50 years and above.

## 3. Results

### 3.1. Data Cleaning

600 women participated in the OPAL trial, however 11 records did not have baseline EQ-5D-3L data and could not be included. From the remaining 589 records, two data subsets were created: (1) ICIQ-UI SF/EQ-5D-3L, and (2) POP-SS/EQ-5D-3L. A total of 12 respondents were removed due to missing data from the ICIQ-UI SF/EQ-5D-3L data subset, leaving a total of 577 respondents included in the analysis with a mean age of 48 years (SD 11.6). From the POP-SS/EQ-5D-3L data subset a total of 34 respondents were removed due to missing data, leaving a total of 555 respondents with a mean age of 47 years (SD 11.5).

### 3.2. EQ-5D-3L

Full health (11111, health state utility value (HRQoL score) = 1.00) using EQ-5D-3L was reported by 267 (46%) respondents. Respondents indicated problems of pain/discomfort (*n* = 218, 38%) and anxiety/depression (*n* = 207, 36%), with fewer indicating problems of usual activities (*n* = 105, 18%), mobility (*n* = 86, 15%), or self-care (*n* = 32, 6%). A negative HRQoL score was calculated for 18 respondents. The worst health state reported was 23233 with a HRQoL score of −0.291 (*n* = 1). The mean total score for the ICIQ-UI SF was just below 13 (SD 3.8), suggesting moderate UI. Item 3 had the highest average score at 6.5 (SD 2.5). The average total score for POP-SS was low at 6.5 (SD 5.6), suggesting mild prolapse symptoms. Table 1 below shows the summary statistics for the EQ-5D-3L and the ICIQ-UI SF and the POP-SS including the individual domain items.

### 3.3. Concurrent Validity of Outcome Measures

Strength and direction of association between EQ-5D-3L and ICIQ-UI SF, and EQ-5D-3L and POP-SS, was tested using Spearman Rank correlation. Table 2 shows that the direction of association for all outcome measures was theoretically valid, with statistically significant negative associations observed for all items in both measures showing that as the severity of UI and severity of prolapse symptoms decrease, quality of life increases. Overall, EQ-5D-3L observed stronger associations with POP-SS, although they were observed to be weak. The correlation coefficients indicated very weak associations between EQ-5D-3L and ICIQ-UI SF.

### 3.4. Known-Groups Validity

The results from the *t*-test for EQ-5D-3L by severity, EQ-5D-3L by age, and EQ-5D-3L by UI Type for each outcome measure are presented in Table 3. Although respondents over 50 years of age did have a lower mean EQ-5D-3L score than those under 50 years of age, this was not found to be a statistically significant difference in health utility. The UI Type was found to be a statistically significant predictor of HRQoL. Those with mixed UI (ICIQ-UI SF (mean = 0.787, SD = 0.288) compared to stress UI (mean = 0.852, SD = 0.225) had a significantly lower EQ-5D-3L score; mean difference 0.065 (95% CI; 0.020, 0.109). UI severity was also found to be a statistically significant predictor of HRQoL as measured using EQ-5D-3L. Those with mild/moderate UI (ICIQ-UI SF: mean = 0.878, SD = 0.188) compared to those with severe UI (mean = 0.750, SD = 0.312) showed statistically significant higher EQ-5D-3L scores; mean difference 0.129 (95% CI; 0.086, 0.171). When severity of prolapse symptoms was defined using POP-SS, the number of respondents per defined group was such that only 74 respondents were defined as “severe”, with a mean HRQoL score of 0.632 (SD 0.335).

## 4. Discussion

Evaluation of the measurement properties of EQ-5D-3L in comparison to ICIQ-UI SF, and to POP-SS, found that overall the EQ-5D-3L was weakly correlated with the POP-SS, a prolapse symptom score, but was only very weakly correlated with the ICIQ-UI SF, albeit in a theoretically valid direction (higher severity of UI or prolapse symptoms associated with a lower quality of life). Analysis of the ability of the EQ-5D-3L to distinguish between groups related directly to the condition of interest found that it was able to distinguish between women with different levels of severity of UI and different types of UI. EQ-5D-3L performed well with regard to the severity of symptoms, discriminating between women with mild/moderate and severe symptoms measured using ICIQ-UI SF, or measured using POP-SS, at a single timepoint.

Choosing among different outcome measures requires knowledge of the quality of instruments, their purpose, reliability and validity. The assessment of the relative performance of OPAL outcome measures highlighted differences between instruments. This is important in understanding whether and how different outcome measures work together. In the absence of other data, it is difficult to offer further explanation on the lack of correlation. However, it is reasonable to suppose that each outcome measure is measuring different constructs with limited confounding factors, and it is common and entirely plausible to have incontinence and a high level of generic health. The OPAL trial did not find evidence that the biofeedback-supported intervention was better than standard pelvic floor muscle training, an important finding for clinicians to appropriately prescribe treatment for UI. However, what is unclear from the trial is to what degree EQ-5D-3L complemented the condition-specific measures to assess the impact of UI and the benefits of the intervention on HRQoL. Given the lack of a gold-standard measure of health utility for UI, in practice trial groups are often defined in terms of clinical measures such as disease severity.

UI is a problem for many women, and interventions to improve this are important from both a clinical and quality of life perspective. Pelvic organ prolapse is a condition which commonly co-exists with UI. RCTs to show the clinical and cost-effectiveness of interventions are vital to develop the most appropriate treatments for UI but also to determine what is a good use of scarce healthcare resources. Traditionally, the EQ-5D is used to calculate QALYs in a cost-utility analysis. The health utility score is calculated using a country-specific value set which reflects general population values about the importance of different aspects of health [21]. The UK value set currently used for EQ-5D is from 1993 and if underlying societal values have changed in relation to expectations about quality of life throughout a person’s lifetime, then using the EQ-5D to calculate a cost per QALY of an intervention could lead to the misallocation of scarce healthcare resources. In the OPAL trial, the number of respondents reporting full health may be indicative of a ceiling effect at baseline which would prevent any improvement in symptoms following the intervention being quantified. In particular, the domains linked to mobility, self-care, and performing usual activities showed 80%, 94% and 82% respectively scored the maximum of 1, indicating no problems in these areas. In comparing this to the domains for pain and anxiety/depression, these showed that those scoring 1 dropped to 62% and 64%, respectively. This may provide an indication as to how UI affects women with fewer issues with regard to performing daily activities and mobility but, potentially, causing more issues with discomfort and mental wellbeing. It may also suggest that the EQ-5D-3L may not be fully responsive and that the newer EQ-5D-5L will likely be more useful in trials of this nature, as it has been designed to take the ceiling effect problem into account.

A limitation of this study is that only cross-sectional data were assessed. For the impact of interventions on HRQoL it is also essential to show that the change in symptoms is being mirrored in a change in quality of life in addition to clinical changes of interest. The extent to which EQ-5D-3L provides complementary information alongside condition-specific measures about change over time requires further assessment. This is important because lack of sensitivity to quantifying the longitudinal benefits of interventions may result in the underestimation of treatment effects.

## 5. Conclusions

Evaluation of the measurement properties of EQ-5D-3L in comparison to ICIQ-UI SF, and in comparison to POP-SS, found EQ-5D-3L is suitable for use in a patient population with stress UI or mixed (stress and urge) UI. In particular, EQ-5D-3L and POP-SS were found to demonstrate concurrent and known-groups validity together. Although EQ-5D-3L and ICIQ-UI SF did not evidence strength of correlation, the direction was empirically consistent and known-groups validity was found. These findings provide useful information to guide researchers in selecting appropriate outcome measures for use in future clinical trials of interventions for female urinary incontinence.

## Figures and Tables

**Table 1:** Summary statistics for ICIQ-UI SF and EQ-5D-3L, POP-SS and EQ-5D-3L.

	N	Mean	Std. Deviation	Min.	Max.
**Urinary Incontinence (ICIQ-UI SF)**					
1. Frequency of urine leakage	577	2.984	1.192	0	5
2. Amount of urine leakage	577	2.887	1.232	0	6
3. Interference with everyday life	577	6.555	2.507	1	10
ICIQ-UI SF Total score	577	12.426	3.844	1	21
**EQ-5D-3L**	577	0.813	0.267	−0.291	1
**Prolapse Symptoms (POP-SS)**					
1. Feeling of something coming down	555	0.816	1.130	0	4
2. Uncomfortable feeling or pain, worse when standing	555	0.577	0.919	0	4
3. Heaviness in lower abdomen	555	0.863	1.056	0	4
4. Heaviness in lower back	555	0.849	1.136	0	4
5. Straining to empty bladder	555	0.766	1.032	0	4
6. Feeling that bladder not completely emptied	555	1.470	1.223	0	4
7. Feeling that bowel not completely emptied	555	1.202	1.197	0	4
POP-SS Total score	555	6.542	5.645	0	28
**EQ-5D-3L**	555	0.816	0.263	−0.291	1

ICIQ-UI SF, International Consultation on Incontinence Questionnaire Urinary Incontinence Short Form; POP-SS, Pelvic Organ Prolapse Symptom Score.

**Table 2:** Spearman Rank Correlation of EQ-5D-3L and ICIQ-UI SF and, EQ-5D-3L and POP-SS.

	Correlation Coefficient	Relationship ^1^
ICIQ-UI Total	−0.298 **	Very weak
ICIQ-UI Item 1	−0.302 **	Weak
ICIQ-UI Item 2	−0.227 **	Very weak
ICIQ-UI Item 3	−0.208 **	Very weak
POP-SS Total	−0.391 **	Weak
POP-SS Item 1	−0.240 **	Very weak
POP-SS Item 2	−0.338 **	Weak
POP-SS Item 3	−0.320 **	Weak
POP-SS Item 4	−0.358 **	Weak
POP-SS Item 5	−0.222 **	Very weak
POP-SS Item 6	−0.289 **	Very weak
POP-SS Item 7	−0.278 **	Very weak

ICIQ-UI SF, International Consultation on Incontinence Questionnaire Urinary Incontinence Short Form; POP-SS, Pelvic Organ Prolapse Symptom Score. ** Correlation is significant at the 0.01 level (2-tailed.). ^1^ Relationship: Very weak (<0.3); Weak (0.3 ≤ r < 0.5); Moderate (0.5 ≤ r < 0.7); Strong (r > 0.7).

**Table 3:** Results for known-groups analysis for EQ-5D-3L/ICIQ-UI SF and EQ-5D-3L/POP-SS.

	N	Mean	S.D.	*df*	*t*	*p*	Mean Difference	95% CI
**EQ-5D-3L/ICIQ-UI SF**								
**Age**								
Under 50	347	0.830	0.257	575	1.954	0.051	0.044	[0.000, 0.089]
Plus 50	230	0.786	0.280					
**UI type**								
Stress UI	227	0.852	0.225	575	2.857	0.004 **	0.065	[0.020, 0.109]
Mixed UI	350	0.787	0.288					
**UI severity ^1^**								
Mild/moderate	282	0.878	0.188	575	5.967	0.000 **	0.129	[0.086, 0.171]
Severe	295	0.750	0.312					
**EQ-5D-3L/POP-SS**								
**Age**								
Under 50	340	0.834	0.249	553	2.021	0.044	0.046	[0.001, 0.091]
Plus 50	215	0.788	0.282					
**UI type**								
Stress UI	217	0.850	0.228	553	2.44	0.015	0.056	[0.011, 0.100]
Mixed UI	338	0.794	0.281					
**Prolapse severity ^2^**								
Mild/moderate	481	0.844	0.238	553	−6.715	0.000 **	0.212	[0.150, 0.274]
Severe	74	0.632	0.335					

CI, confidence interval; *df*, degrees of freedom; ICIQ-UI SF, International Consultation on Incontinence Questionnaire Urinary Incontinence Short Form; POP-SS, Pelvic Organ Prolapse Symptom Score; S.D., standard deviation; UI, urinary incontinence ** Significant at the 0.01 level (2-tailed). ^1^ ICIQ-UI SF total score < 13 mild/moderate. ^2^ POP-SS total score < 14 mild/moderate.

## Data Availability

Any data sharing request should be submitted to the corresponding author.
